# Inhibitory activity of a green and black tea blend on *Streptococcus mutans*

**DOI:** 10.1080/20002297.2018.1481322

**Published:** 2018-06-05

**Authors:** Helena Barroso, Rita Ramalhete, Ana Domingues, Samanta Maci

**Affiliations:** a Laboratório de Microbiologia Aplicada Egas Moniz (LMAEM), Caparica, Portugal; b Centro de Investigação Interdisciplinar Egas Moniz, ISCSEM, Caparica, Portugal; c Kemin Health, a division of Kemin Foods L.C. Lisbon, Portugal

**Keywords:** Antimicrobial activity, oral health, *S. mutans*, aqueous extract, MIC, MBC

## Abstract

Through the years, tea consumption has been associated with good health, and some publications are related to oral health. The bioactive components of green tea are thought to be able to influence the process of caries formation through inhibition of proliferation of the streptococcal agent, interference with the process of bacterial adhesion to tooth enamel, and inhibition of glucosyl transferase and amylase; however, little is known about black tea and oral health. The aim of the present *in-vitro* study was to determine the inhibitory activity of a novel, patent-pending and proprietary blend of green and black tea aqueous extracts on *Streptococcus mutans*, a bacterium widely associated with plaque development and tooth decay. A minimum inhibitory concentration (MIC) of 12.5 mg/mL and a minimum bactericidal concentration (MBC) of 12.5 mg/mL was established against *S. mutans*, meaning that at concentrations of 12.5 mg/mL and higher, the proprietary tea blend is effective against the growth of *S. mutans*. This MIC concentration is lower than the ones reported in the literature for alcoholic black tea and green tea extracts tested separately. As a promising natural ingredient for oral health, this finding is a good indicator for the use of this proprietary blend of black and green tea water extracts.

Aside from water, tea is the most commonly consumed beverage in the human diet [1]. Tea is obtained from processing the tender leaves and buds of the *Camellia sinensis* plant. Depending on the manufacturing process different types of tea are obtained. Green tea is obtained by withering the *C. sinensis* leaves to remove moisture followed by steam treatment to inactivate enzymes in the tea leaves [1]. Green tea is characterized by the presence of non-oxidized phenolic compounds named catechins, which are the mainly responsible for the antioxidant capacity of green tea [2,3]. In black tea, *C. sinensis* leaves are first withered to remove moisture and then rolled and allowed to ferment. This process results in the oxidation of the leaves by enzymes present in the tea leaves and conversion of the catechins to higher molecular weight polyphenolic compounds such as theaflavins and thearubigins that give black tea the distinctive colour and flavor [1]. Non-catechin components, such as tannins and thearubigins, are the main components responsible for the antioxidant activity of black tea [2].

Polyphenols are an important class of active secondary metabolites of plants that present activity against several pathogens. This antibacterial capacity, both against Gram-positive and Gram-negative bacteria, can be explained by different mechanisms such as the interaction with bacterial proteins and cell-wall structures, the damage of cytoplasmic membranes, the reduction of membrane fluid, the inhibition of nucleic acid synthesis, cell wall synthesis or energy metabolism [reviewed in 4].

Through the years, tea consumption has been associated with good health. Many published studies have established correlations between the intake of green and black tea and several benefits mainly linked to the reduction of oxidative stress and inflammation reviewed in [reviewed in 1,5–8]. Tea benefits can also be linked to skin health, weight management and muscle recovery [9–12].

Other associated health benefits such as those related to oral health have also been reported. Populations that consume high quantities of polyphenol-rich foods or beverages have low caries incidence and a better overall oral health [13]. Besides cleaning of the teeth, the use of natural compounds with antimicrobial effect to limit the growth of cariogenic microorganisms in the oral cavity could also contribute to prevention of caries.

The oral cavity is one of the most microbially colonized parts of our bodies, harboring distinct habitats that support different microbial communities. These constitute an important link between oral and general health. These microorganisms have both pro- and antiinflammatory activities that are crucial for maintaining homeostasis [14]. Almost all the bacteria in the healthy oral cavity belong to the Firmicutes, Actinobacteria, Proteobacteria, Fusobacteria, Bacteroidetes and Spirochaetes phyla [15].

In a healthy mouth, the microbial communities are stable, but biological changes in our body can affect the balance of the species within these communities, leading to an increase of cariogenic or periodontopathic bacteria [14].

One of the most relevant pathogens linked to the formation of dental plaque and the development of caries is *Streptococcus mutans*.

The search for natural products that inhibit the growth of cariogenic bacteria has been persuaded, in order to substitute the current antiseptics used, due to their undesirable effects [16,17].

Different studies have shown that tea presents an inhibitory activity against *S. mutans* [18,19]. *In-vivo* and *in-vitro* studies have reported a reduction in bacterial counts, saliva/plaque pH and gingivitis associated with the local administration of green tea extracts [20–25], and one study has shown the benefits of black tea administration [26]. To our knowledge, no study has explored the potential benefit of a combination of green and black tea.

The bioactive components of green tea are able to influence the process of caries formation through different mechanisms: they may inhibit proliferation of the streptococcal agent, interfere with the process of bacterial adhesion to tooth enamel, and act as inhibitors of glucosyl transferase and amylase [19,27,28]; however, little is known about black tea and oral health.

Based on these published studies, an *in-vitro* test was designed to determine the inhibitory activity of a novel, patent-pending, proprietary blend of green and black tea extracts on *S. mutans*, a bacterium widely associated with plaque development and tooth decay. Our goal was to verify if the association between green and black tea would show antibacterial activity against this cariogenic bacterium.

## Study product

The study product is a proprietary blend of green and black tea extracts, AssuriTEA® (Kemin Foods, Des Moines, IA). AssuriTEA is a high quality 100% aqueous extract of *Camellia sinensis* leaves harvested from a plant selected for their natural high polyphenols content. The extract contains at least 40% total polyphenols with a minimum of 20% catechins and theaflavins combined.

The manufacture of AssuriTEA involves the blending of food-grade, black and green tea extracts produced from dried black and green tea leaves (both *C. sinensis L*.) through gentle hot water extraction followed by spray drying. The powder blend of the two extracts is then further processed through a roller compaction process to produce a water dispersible form of finished powder.

The 100% hot-water-extraction process performed without the use of any processing aids during the extraction produces a safe (like traditional green tea infusions) and naturally occurring polyphenol profile and avoids the elimination of other important nutrients, such as vitamins and minerals, which also may contribute to beneficial health effects of brewed tea. Qualitatively, this process does not impart any chemical modification to the naturally occurring components in the black and green tea.

This proprietary ingredient has been shown, in a randomized double-blind placebo-controlled study conducted in healthy male subjects, to be well tolerated and increase the serum antioxidant status in participants [29].

AssuriTEA was tested in the following six concentrations: 25, 12.5, 6.25, 3.13, 1.56 and 0.78 mg/mL (prepared in sterile water). Each concentration was tested in triplicate.

## Bacterial strain

The microorganism used was the reference strain *S. mutans* ATCC 35,668 grown in blood agar (Columbia agar + 5% sheep blood, Biomérieux) at 37ºC under anaerobic conditions.

## Microbiological assays – determination of minimum inhibitory concentration (MIC) and minimum bactericidal concentration (MBC)

All the *in-vitro* inhibition tests were performed according to CLSI (Clinical and Laboratory Standards Institute) guidelines [30]. Growth of the reference strain *S. mutans* ATCC 35,668 was always accessed in the different media used.

The MIC was determined by the agar-dilution test, in plates of blood agar (Columbia agar + 5% sheep blood, Biomérieux) with the different concentrations of the test products incorporated. Briefly, the reference strain, *S. mutans* was diluted in Brain Hearth Infusion Broth (BHI) (Biokar Diagnostics) to make a test suspension containing about 1.5 × 10^6^ CFU/mL.

Solutions of the AssuriTEA blend were prepared in sterile water in the following six concentrations: 25, 12.5, 6.25, 3.13, 1.56 and 0.78 mg/mL.

One milliliter of each tea solution was incorporated into melted agar (15 mL) supplemented with sheep blood (5%), which was then poured onto sterile petri dishes and allowed to solidify.

When solidified, the *S. mutans* was inoculated onto the surface of the blood agar plates, with a swab as a central line, and the plates were incubated for 24 h at 37°C.

The MBC was determined by the broth dilution test followed by the spread of the test solutions onto blood agar medium plates [30]. Briefly, the reference strain, *S. mutans* was diluted in BHI, to make a test suspension containing about 1.5 × 10^6^ CFU/mL.

Solutions of the AssuriTEA blend were prepared in sterile water in the following concentrations: 50, 25, 12.5, 6.25, 3.13 and 1.56 mg/mL. In sterile tubes, 1 mL of tea solution was added to 1 mL of culture suspension. The resultant tea concentrations in each of the tubes were, hence, 25 mg/mL, 12.5 mg/mL, 6.25 mg/mL, 3.13 mg/mL, 1.56 mg/mL and 0.78 mg/mL. Three positive controls (1 mL water + 1 mL prepared culture) and three replicates for each concentration of AssuriTEA blend were also prepared. These solutions were incubated for 24 h at 37°C. After that, they were used to determine the MBC: 100 µL of each of these solutions was spread onto blood agar medium plates and incubated for 24 h at 37°C. Due to its turbidity it was not possible to confirm the MIC values determined with the agar dilution test explained before.

**Figure 1.**
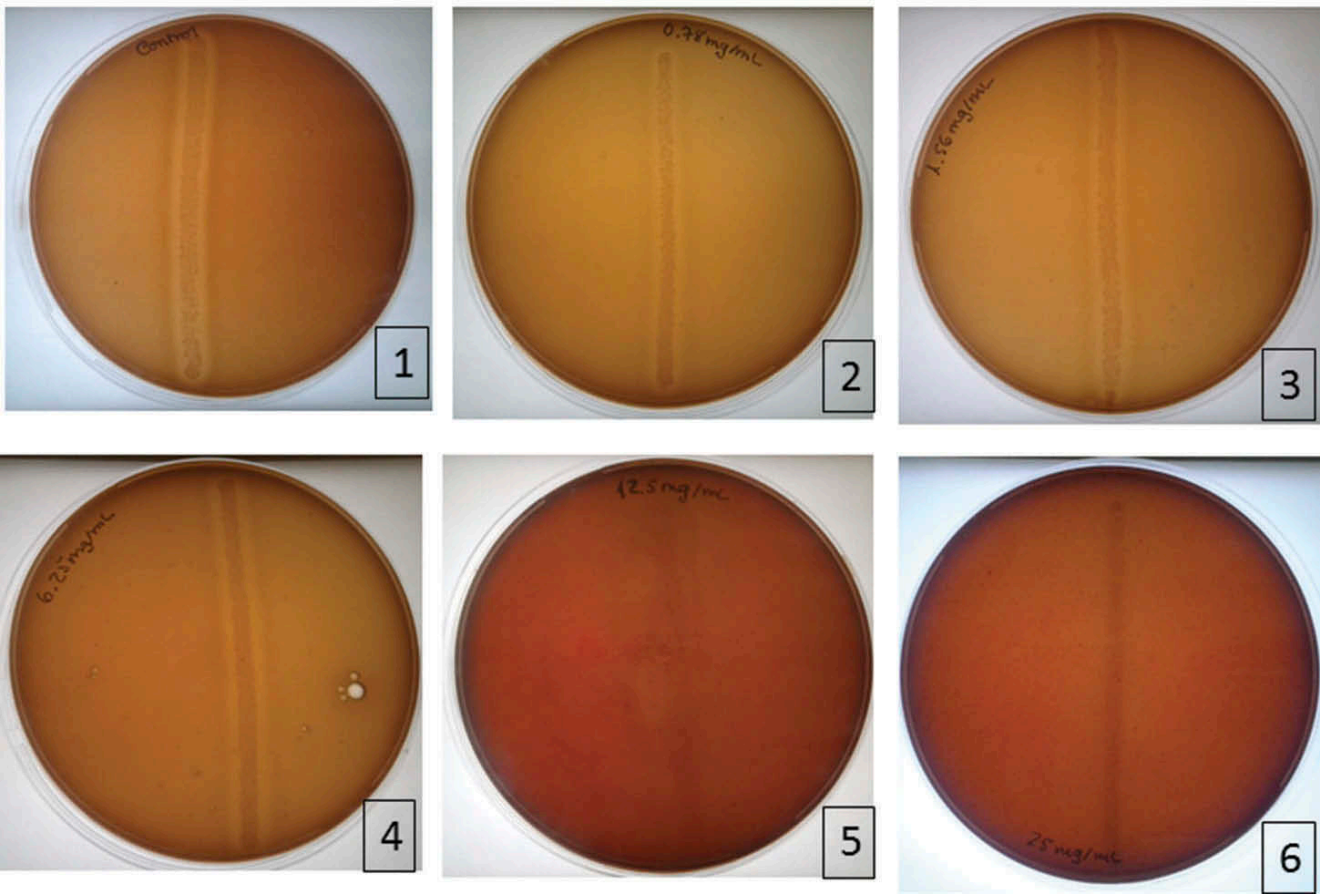
Results of MIC determination in blood agar. 1. Positive control; 2. Plate with 0.78 mg/mL; 3. Plate with 1.56 mg/mL; 4. Plate with 6.25 mg/mL; 5. Plate with 12.5 mg/mL; 6. Plate with 25 mg/mL.

The MIC, or minimum concentration where no growth of *S. mutans* was observed, was found to be 12.5 mg/mL. These results are shown in Figure 1.

**Figure 2.**
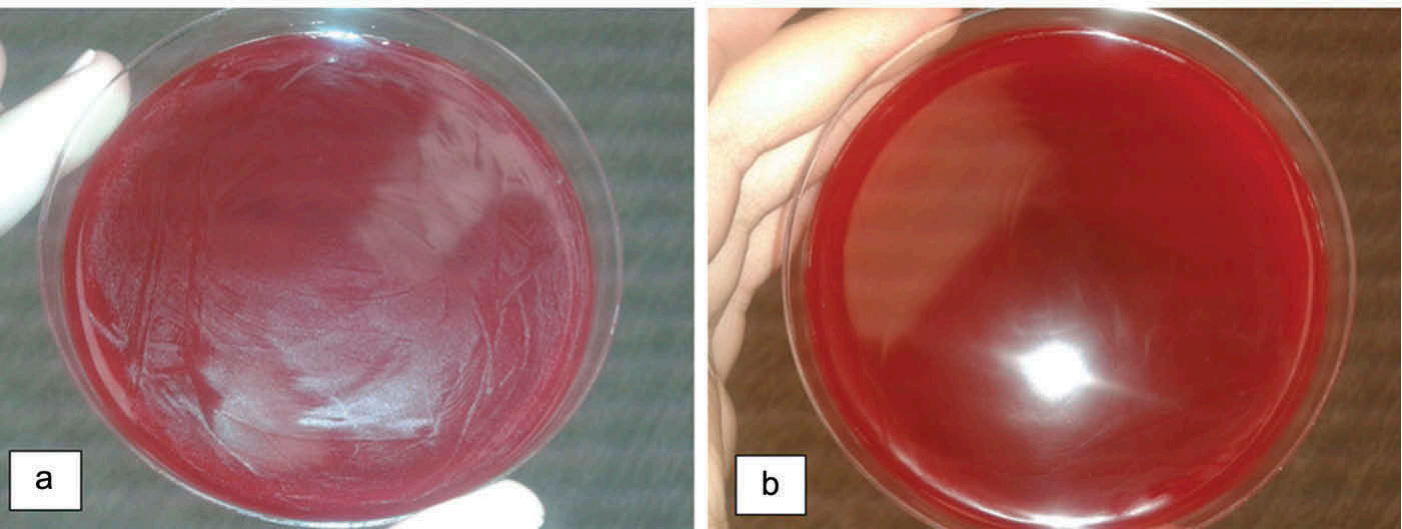
Blood agar inoculated with solutions of AssuriTEA corresponding to (a) 0.78 mg/mL and (b) 12.5 mg/mL.

After spread onto blood agar, a decrease in the bacterial growth was seen for the sequentially higher concentrations, and the minimum concentration showing no growth of *S. mutans*, for the AssuriTEA blend, corresponded to 12.5 mg/mL, being the determined MBC (Figure 2). For AssuriTEA, a MIC of 12.5 mg/mL and a MBC of 12.5 mg/mL were established against *S. mutans*, meaning that at concentrations of 12.5 mg/mL and higher, AssuriTEA is effective against the growth of *S. mutans*, a bacterium commonly associated with the formation of dental plaque and tooth decay.

In this study, it was shown that AssuriTEA presented an inhibitory action against *S. mutans* when applied at a level of 12.5 mg/mL or higher.

For thousands of years, plants have been used in medicine. The interest in naturally derived biological compounds that may have the potential to be used as therapeutics in medicine and dentistry is rising [13,31]. There are previous published studies on the activity of tea components against bacteria responsible for oral diseases. Some research groups showed that tea extracts inhibited the attachment of bacteria to oral surfaces [32] and are effective against cariogenic microorganisms [23]. It was observed that rinsing the mouth with green tea led to a significant reduction of cariogenic bacteria like *S. mutans* and *Lactobacillus* [19,33]. However, most of these studies were focused on the action of isolated tea components, namely polyphenols.

Since tea is consumed regularly in an aqueous form (tea infusion), this study was carried out to determine the effect of aqueous extracts of tea on *S. mutans* growth. It was demonstrated that the growth of *S. mutans* was inhibited at a concentration of 12.5 mg/mL and higher. This MIC concentration is lower than the ones reported by Naderi et al. [26], of 50 mg/mL for alcoholic black tea extract and 150 mg/mL for alcoholic green tea extract tested separately. Although there are other studies where a lower MIC was obtained (but with organic extracts) [20], this concentration of AssuriTEA was bactericidal.

*S. mutans* is one of the most important cariogenic bacteria as it ferments different sugars and is tolerant to acidic environments. The bacterium’s growth and metabolism promote changes in the oral environment, enabling other bacteria to also colonize the teeth leading to the formation of dental biofilm. Cariogenic bacteria exist naturally in the mouth, so it is essential to prevent their growth and colonization. Reducing the intake of sugars, brushing, flossing and the use of antimicrobial mouth rising solutions can help to control the growth of cariogenic bacteria [14,34]. The use of natural products with antibacterial action, such as AssuriTEA, is also an option.

In conclusion, AssuriTEA, the blend of green and black tea aqueous extract, showed bactericidal activity *in vitro* against *S. mutans*, demonstrating the ability to control the growth of this bacterium. This is the first study that investigates the antibacterial activity of such a mixture. Although this was an *in-vitro* study, and only one cariogenic bacteria was used, this finding is a good indicator for the use of this proprietary blend of black and green tea extract as a promising natural ingredient for oral health.
